# A Year's Experience with Three New Remedies

**Published:** 1894-11

**Authors:** I. N. Carr

**Affiliations:** Tarboro, N. C.


ARTICLE VII.

### A YEAR'S EXPERIENCE WITH THREE NEW REMEDIES.

BY DR. I. N. CARR, TARBORO, N. C.

A year ago I demonstrated before this Society the use of three preparations that had but recently been brought to the attention of the profession by Dr. E. C. Kirk, of Philadelphia, and by invitation of the President of the Virginia Association, I attended their meeting at Charlottesville, and did the same thing, and promised to present the results of my experience at a subsequent meeting. I now present for your consideration this report. which is a practical result of a fair test of their virtues, and which, I trust, will be of interest to you all.

The preparations are those of pyrozone, sodium peroxid, and Dr. Schrier's Compound of Sodium and Potassium, in metallic form. Each of these have their places in my daily practice, and it requires only ordinary judgment to decide which to use in each case, or whether it is best to use one or more. As, for instance, if you are going to treat a tooth for a "blind abscess," adjust the rubber-dam, and spray the cavity and canals with five per cent. etherial pyrozone, allowing no instrument to enter the root. After discharge of pus ceases, spray the root with the three per cent. aqueous solution; then use the sodium peroxid, or the sodium and potassium. If it should be a lower molar, I prefer the sodium peroxid, because the roots of these teeth are so thin, and the canals so small, that it is rather difficult to carry a broach loaded with the sodium and potassium to the ends of the roots; but with the other preparation I put a few drops of a fifty per cent. solution into the cavity; and by means of a fine broach work it clear down to the end of the root, and if a little passes beyond, it does no harm. Now wash the roots with warm water that has first been boiled, dry with absorbent cotton and the hot-air syringe, and fill with what you can make the best root filling of. I do not hesitate to treat and fill such cases at one sitting, though if the patient lives in town I sometimes seal the root hermetically till the next day, particularly if there has been periostial disturbance, but ordinarily I fill at one sitting. If the tooth is an upper front, after evacuating the abscess by the five per cent. pyrozone, I use the preparation of sodium and potassium of Dr. Schrier's. This will destroy all germs of animal or vegetable life, and leaves the root sweet and clean; but during the past six months I have used the sodium peroxid (known under the formula $N2O2$) more than anything else; it is much cheaper, bleaches the teeth nicely, and does the work just as well.—*Items of Interest.*

